# Anticholinergic Burden Rationalisation Before Dementia Treatment in an Old Age Psychiatry Community Team

**DOI:** 10.1192/bjo.2025.10544

**Published:** 2025-06-20

**Authors:** Isaac Alakeji, Aruna Ravishankar, Siobhan Jeffery

**Affiliations:** Devon Partnership NHS Trust, Exeter, United Kingdom

## Abstract

**Aims:** To carry out a retrospective audit of medical records of people with newly diagnosed dementia in North Devon Community Older People’s Mental Health Team for any evidence of anticholinergic burden review before dementia treatment.

**Methods:** Data for 49 patients with dementia were identified based on evidence of clinical coding on SystmOne (the Trust’s new electronic records system) from November 2023 to August 2024. Most of the newly diagnosed patients with dementia were not captured due to missing clinical coding.

Electronic records, including GP referral letters, assessment notes, and MDT discussions, were reviewed to determine whether anticholinergic medicines were rationalised and whether ACB scores were recorded before initiating medication for dementia.

In addition, pre-referral medications were reviewed from the GP referral letters to establish pre-referral anticholinergic burden and ACB scores.

Values from the ACB calculator were used for anticholinergic burden estimation in this audit as it collates information from the German Anticholinergic Burden Scale and the Anticholinergic Cognitive Burden Scale, which have been demonstrated to have the highest validity and reliability. According to the NICE guideline [NG97], there is not sufficient evidence to recommend one validated tool over the others.

**Results:** Out of 49 patient records, 27 were included in this audit. Twenty-two were excluded due to not meeting the inclusion criteria.

None of the clinicians documented that anticholinergic medicines were rationalised, and only 2 (7.4%) documented that the patient’s current medications were reviewed.

There was no documentation of ACB score for any of the patients included in this audit; whereas, the vast majority (70.4%) of them were on regular medications with anticholinergic burden before presentation and such medications were prescribed for their anticholinergic effect in 1 out of 10 (11.1%) of cases.

More than half (52.6%) of the audit patients with pre-referral anticholinergic burden had ACB scores of 3 or higher (high risk).

The most commonly prescribed medications leading to raised ACB were metformin, lansoprazole and sertraline in descending order of frequency.

Those prescribed specifically for their anticholinergic effect were solifenacin and oxybutynin with ACB score of 3 each.

**Conclusion:** We are not documenting that we rationalised anticholinergic medicines before initiating anti-dementia treatment.

Almost 3 out of 4 of the patients referred to our team for dementia diagnosis were on medications with an anticholinergic burden. More than half of those on anticholinergic medications had ACB scores in the high-risk range.

The most commonly prescribed medication resulting in anticholinergic burden was metformin.

